# Inhibition of ferroptosis improves developmental competence of vitrified–warmed oocytes

**DOI:** 10.3389/fendo.2026.1851814

**Published:** 2026-06-15

**Authors:** Tianli Huang, Yutong Huang, Xuanqi Liu, Jiaying Mo, Yining Cao, Yishang Yan, Jun Ren, Xinyuan Li, Jianzhong Sheng, Hong Zhu, Hefeng Huang

**Affiliations:** 1 The Key Laboratory of Reproductive Genetics (Zhejiang University), Ministry of Education, Zhejiang University, Hangzhou, China; 2 Assisted Reproduction Unit, Department of Obstetrics and Gynecology, Sir Run Run Shaw Hospital, School of Medicine, Zhejiang University, Key Laboratory of Reproductive Dysfunction Management of Zhejiang Province, Hangzhou, China; 3 The Second Affiliated Hospital, School of Medicine, Zhejiang University, Hangzhou, China; 4 Institute of Reproduction and Development, Shanghai Key Laboratory of Reproduction and Development, Obstetrics and Gynecology Hospital, Fudan University, Shanghai, China; 5 Department of Obstetrics and Gynecology, Center for Reproductive Medicine, the Fourth Affiliated Hospital of School of Medicine, and International School of Medicine, International Institutes of Medicine, Zhejiang University, Yiwu, China; 6 Obstetrics and Gynecology Hospital, Institute of Reproduction and Development, Shanghai Ji Ai Genetics and IVF Institute, Fudan University, Shanghai, China

**Keywords:** embryo development, ferroptosis, oocyte, oxidative stress, vitrification

## Abstract

**Background:**

Oocyte vitrification is widely used for female fertility preservation. However, current vitrification procedures can compromise oocyte quality, leading to increased oxidative stress and organelle dysfunction. Therefore, optimizing vitrification strategies to improve oocyte developmental competence is essential.

**Methods:**

Mouse oocytes were randomly assigned to control (CON) and vitrification (VIT) groups. After storage in liquid nitrogen for over a month, vitrified oocytes were warmed and subjected to intracytoplasmic sperm injection (ICSI). Subsequent embryonic development was assessed *in vitro*. Transcriptomic and proteomic analyses were performed to identify differentially expressed genes and proteins, followed by validation using quantitative real-time PCR, immunofluorescence, and functional assays with the ferroptosis inhibitor ferrostatin-1 (Fer-1) and glutathione monoethyl ester (GSH-MEE).

**Results:**

Vitrification–warming impaired oocyte quality and reduced embryonic developmental competence in mouse oocytes. Vitrified–warmed oocytes exhibited mitochondrial dysfunction, increased oxidative stress, and enhanced lipid peroxidation. In addition, ferroptosis-related signaling was activated after vitrification–warming. Treatment with Fer-1 or GSH-MEE alleviated oxidative stress, improved mitochondrial function, and partially restored intracellular redox balance and iron homeostasis. Notably, both treatments significantly improved the developmental potential of vitrified–warmed oocytes.

**Conclusions:**

Our findings suggest that vitrification-warming is associated with ferroptosis-related alterations in mouse oocytes. Inhibition of ferroptosis by Fer-1 or GSH-MEE partially improved oocyte quality and preimplantation embryonic development after cryopreservation.

## 1 Introduction

The global decline in birth rates, coupled with delayed childbearing, has become an increasingly prominent public health concern (1–3). With the advancement of assisted reproductive technology (ART), oocyte cryopreservation has emerged as an important strategy for female fertility preservation. Currently, oocyte cryopreservation is widely applied in ART, particularly for patients who are unable to obtain sufficient sperm on the day of oocyte retrieval or for women who wish to postpone childbearing for social or medical reasons (4, 5). In addition, with improving survival rates among cancer patients, many women choose to preserve their fertility by cryopreserving oocytes prior to chemotherapy or radiotherapy (6).

Cryopreservation enables long-term storage of genetic material and is extensively utilized in human assisted reproduction. Since the first successful birth from a cryopreserved oocyte using slow freezing in 1986, substantial progress has been made in this field (7–9). Compared with slow freezing, vitrification minimizes ice crystal formation and reduces cellular damage, thereby significantly improving fertilization, developmental, and implantation outcomes (10–12). Consequently, vitrification has largely replaced slow freezing as the standard method for oocyte cryopreservation in clinical practice. However, accumulating evidence indicates that vitrification–warming is associated with increased reactive oxygen species (ROS) production, lipid peroxidation, decreased ATP levels, reduced glutathione (GSH) content, diminished GPX4 activity, and organelle dysfunction (13–17).

Ferroptosis is a regulated form of cell death characterized by iron-dependent lipid peroxidation, depletion of glutathione, and inactivation of glutathione peroxidase 4 (GPX4) (18, 19). Cells undergoing ferroptosis exhibit mitochondrial dysfunction and redox imbalance, leading to excessive ROS accumulation and oxidative damage to membrane phospholipids (20). Recent studies have shown that supplementation with antioxidants, such as melatonin, astaxanthin, and glutathione, during vitrification or after warming can enhance oocyte antioxidant capacity and improve developmental potential (16, 21, 22). Moreover, ferroptosis has been implicated in a wide range of pathological processes, including neurodegenerative diseases, cancer, and cardiovascular disorders (19, 23). However, whether ferroptosis contributes to oocyte damage during vitrification–warming remains unclear.

In the present study, we established a mouse model of oocyte vitrification–warming and demonstrated that vitrified–warmed oocytes exhibit ferroptosis-related alterations, including increased ROS and Fe^2+^ levels, enhanced lipid peroxidation, and decreased GPX4 expression and GSH content. Importantly, supplementation with the ferroptosis inhibitor ferrostatin-1 (Fer-1) or glutathione monoethyl ester (GSH-MEE) mitigated these effects, improved oocyte quality, and enhanced embryonic developmental competence.

## 2 Materials and methods

### 2.1 Animals

All animal procedures were conducted in accordance with protocols approved by the Institutional Animal Care and Use Committee of Zhejiang University. Experimental animals were obtained from Vital River Laboratory Animal Technology Co., Ltd. and the Laboratory Animal Center of Zhejiang University. Mice were housed under controlled conditions (24 °C, 12 h light/dark cycle) with ad libitum access to food and water throughout the study.

### 2.2 Oocyte collection

Oocyte collection was performed as previously described (24). Briefly, female C57BL/6N mice (6–8 weeks old) were intraperitoneally injected with 5 IU pregnant mare serum gonadotropin (PMSG; Nanjing Aibei Biotechnology Co., Ltd., M2630). After 46–48 h, 5 IU human chorionic gonadotropin (hCG; Nanjing Aibei Biotechnology Co., Ltd., M2530) was administered to induce superovulation. Oviducts were collected 13–14 h after hCG injection, and ampullae were punctured to release cumulus–oocyte complexes (COCs). Cumulus cells were removed using 0.3 mg/mL hyaluronidase (H4272; Sigma), followed by repeated washing in M2 medium (M7167; Sigma-Aldrich).

### 2.3 Oocyte vitrification and warming

Oocyte vitrification and warming were performed as previously described with minor modifications (9, 25). The Cryotop method was used as described in previous studies (9). Commercial vitrification and warming kits (Kitazato Biopharma Co., Ltd., Japan) were used according to the manufacturer’s instructions. Briefly, oocytes were first equilibrated in equilibration solution for 5 min, then transferred to vitrification solution. Subsequently, oocytes were loaded onto Cryotop carriers and plunged into liquid nitrogen within 1 min.

For warming, vitrified oocytes were rapidly transferred from liquid nitrogen into warming solution for 1 min, followed by incubation in dilution solution for 3 min and two washes in washing solution for 5 min each. Oocytes with intact zona pellucida and plasma membranes were considered viable. Surviving oocytes were cultured in KSOM medium (Sigma-Aldrich, USA; MR-101) at 37 °C in a humidified incubator with 5% CO_2_ for 3 h before further experiments.

### 2.4 Intracytoplasmic spermatozoa injection

Epididymal spermatozoa were collected from the cauda epididymis of 10–12-week-old C57BL/6N mice and incubated in HTF medium (Sigma-Aldrich, USA; MR-070) for 30 min at 37 °C under 5% CO_2_. Subsequently, 1 µL of sperm suspension was mixed with 10 µL of 10% polyvinylpyrrolidone (PVP) in HEPES-buffered CZB medium in an ICSI chamber. ICSI was performed as previously described (26). Briefly, sperm heads were separated from tails using piezo pulses applied to the neck region and immediately injected into vitrified–warmed oocytes. After a 10 min recovery period at room temperature, oocytes were washed at least three times and transferred to KSOM medium. After 1 h of recovery, oocytes were considered to have survived when the zona pellucida and plasma membrane remained intact, the perivitelline space appeared clear and of normal size, and no signs of cytoplasmic leakage, oocyte shrinkage, cytoplasmic darkening, or granulation were observed. In addition, there was minimal or no visible separation between the zona pellucida and the oocyte cytoplasm (27). Fertilization was assessed by the presence of two pronuclei (2PN) at 6 h post-ICSI. Embryo development was evaluated by recording the formation rates of 2-cell, 4-cell, morula, and blastocyst stages at 24, 48, 72, and 96 h post-ICSI, respectively.

### 2.5 Drug treatment

RSL3 (Selleck, USA;S8155) was diluted at a final concentration of 3 μM for 3 h in KSOM medium under mineral oil at 37 °C in an atmosphere of 5% CO_2_. The concentration of RSL3 was selected based on previous reports (28). Control oocytes were cultured under the same conditions without RSL3. After treatment, oocytes were subjected to the assessment of intracellular ROS, Fe^2+^ accumulation, lipid peroxidation, mitochondrial membrane potential, intracellular GSH levels, and GPX4 immunofluorescence. The remaining oocytes were used for intracytoplasmic sperm injection (ICSI) and subsequent embryo culture to evaluate developmental competence.

Glutathione monoethyl ester (GSH-MEE; Sigma-Aldrich, USA; G1404) was diluted in KSOM medium to a final concentration of 0.5 mM. The concentration of GSH-MEE was selected based on previous reports (24). Ferrostatin-1 (Fer-1; Sigma-Aldrich, USA; SML0583) was dissolved in DMSO to prepare a 10 mg/mL stock solution and subsequently diluted in KSOM medium to the desired working concentrations.

For *in vitro* rescue assays, control (CON) and vitrification (VIT) groups were treated with equal volumes of KSOM medium without Fer-1 or GSH-MEE. The VIT group served as the vehicle control and received the same concentration of DMSO as used for Fer-1 preparation. The final DMSO concentration was identical in all DMSO-containing groups. CON + Fer-1 and CON + GSH-MEE groups were included to assess the effects of pharmacological treatment on non-vitrified oocytes. Fresh and vitrified–warmed oocytes were cultured for 3 h in KSOM medium with or without Fer-1 or GSH-MEE, followed by intracytoplasmic sperm injection (ICSI). Embryonic development was monitored to the blastocyst stage, and the effects of Fer-1 or GSH-MEE supplementation were evaluated.

### 2.6 Transcriptome RNA library construction and sequencing

Transcriptomic analysis of MII oocytes was performed using the Smart-seq2 protocol. Two independent biological replicates were included for each group, with 40 oocytes per sample. Following cell lysis and cDNA amplification, amplified cDNA was purified and used for library construction. Library preparation and sequencing were conducted as previously described (29). Differential expression analysis was carried out using DESeq2. P values were adjusted for multiple testing using the Benjamini–Hochberg method. Genes with an absolute fold change > 2 and a false discovery rate (FDR) < 0.05 were considered differentially expressed.

### 2.7 Proteomic sample preparation

For proteomic analysis, 30–50 oocytes were collected per group. The zona pellucida was removed using pre-warmed acidic Tyrode’s solution (Sigma-Aldrich, USA; MR-004), followed by washing three times in 1× PBS. Oocytes were then lysed in buffer containing 1% sodium deoxycholate, 10 mM TCEP, and 40 mM 2-chloroacetamide in 20 mM Tris–HCl (pH 8.5), boiled for 10 min, and sonicated to denature proteins and shear DNA. Proteins were digested with trypsin and LysC at a 1:100 enzyme-to-protein ratio and subsequently desalted using SDB-RPS StageTips. Eluted peptides were dried in a SpeedVac and resuspended in 0.1% formic acid for mass spectrometry analysis.

### 2.8 LC-MS/MS analysis

LC–MS/MS analysis was performed using an UltiMate™ 3000 RSLCnano system coupled to a Q Exactive HF-X mass spectrometer. Peptides were loaded onto a trap column (75 µm × 20 mm, 3 µm C18, 100 Å; Thermo Fisher Scientific) at a maximum pressure of 620 bar using mobile phase A (0.1% formic acid in H_2_O). Peptide separation was carried out on an analytical column (75 µm × 500 mm, 3 µm C18, 100 Å; Thermo Fisher Scientific) with a gradient of 4–60% mobile phase B (80% acetonitrile, 0.08% formic acid) at a flow rate of 250 nL/min for 280 min. Mass spectrometry data were acquired in data-dependent acquisition (DDA) mode, with one full MS scan (m/z 300–1800, resolution 60,000 at m/z 200, AGC 3 × 10^6^), followed by 40 MS/MS scans using high-energy collision dissociation (AGC 1 × 10^5^, maximum injection time 100 ms, isolation window 1.6 m/z, normalized collision energy 27%).

### 2.9 Processing of raw LC–MS/MS data

Raw LC–MS/MS data were processed using MaxQuant software (version 1.6.2.10) (30) and searched against the mouse UniProtKB database. Full tryptic specificity was required, allowing up to two missed cleavages. Carbamidomethylation (C) was set as a fixed modification, while oxidation (M) and N-terminal acetylation were set as variable modifications. Mass tolerance was set to 10 ppm for MS scans acquired in the Orbitrap analyzer. The false discovery rate (FDR) was set to 1% at both protein and peptide levels and estimated using a reverse database strategy. Protein quantification was performed using the iBAQ algorithm, and data were log_2_-transformed for downstream analysis. Two independent biological replicate batches were included for each group. Differentially expressed proteins were identified using an absolute fold change > 1.5 and an FDR-adjusted *P* value < 0.05. The proteomics analysis results are provided in **Supplementary Table 2**.

### 2.10 RNA isolation and quantitative real-time PCR

A total of 40 oocytes were collected for RNA extraction using the Dynabeads™ mRNA DIRECT Kit (Thermo Fisher Scientific, Cat. 61012) according to the manufacturer’s instructions. RNA concentration and purity were assessed using a NanoDrop ND-1000 spectrophotometer (NanoDrop, Wilmington, DE, USA). cDNA was synthesized from the extracted RNA using HiScript II RT SuperMix (Vazyme, Nanjing, China, Cat. R222-01) according to the manufacturer’s instructions. Quantitative real-time PCR was performed using TB Green Premix Ex Taq on a LightCycler 480 II system (Roche Diagnostics, Basel, Switzerland). The PCR conditions were as follows: 95 °C for 30 s, followed by 40 cycles of 95 °C for 5 s and 60 °C for 30 s. Melting curve analysis confirmed the specificity of the amplified products. Relative gene expression levels were calculated using the 2^−ΔΔCt method, with Gapdh as the internal control. No-template controls were included in each qPCR run. Primers were synthesized by Sangon Biotech (Shanghai, China), and primer information is provided in **Supplementary Table 1**.

### 2.11 Immunofluorescence

Immunofluorescence staining was performed according to a standard protocol. Briefly, oocytes were fixed in 4% (w/v) paraformaldehyde (PFA) for 30 min at room temperature, followed by permeabilization in 0.5% Triton X-100 in PBS for 20 min. Oocytes were then blocked in blocking buffer (3% bovine serum albumin, 0.1% Tween 20, and 0.01% Triton X-100 in PBS) for 1 h at room temperature, and all antibodies were diluted in the same buffer. Isotype-matched IgG controls were included in parallel to verify antibody specificity.

Oocytes were incubated with an anti-GPX4 primary antibody (ab125066, Abcam; 1:200) at 4 °C overnight. After washing three times with 0.1% Tween 20 in PBS (5 min each), oocytes were incubated with appropriate secondary antibodies for 1 h at room temperature. Nuclei were stained with 4′,6-diamidino-2-phenylindole (DAPI) for 15 min. Oocytes were then washed three times (10 min each), mounted on glass slides for imaging. At least 30 oocytes per group were analyzed across three independent replicates. Images were acquired using a Zeiss LSM880 confocal microscope, and fluorescence intensity was quantified using ImageJ software (NIH, Bethesda, MD, USA).

### 2.12 Fluorescence staining and confocal microscopy

For mitochondrial staining, oocytes were incubated in M2 medium containing 200 nM MitoTracker Green FM (40742ES50, Yeasen) for 30 min at 37 °C under 5% CO_2_ in the dark. After three washes in fresh M2 medium (5 min each), oocytes were mounted on glass-bottom dishes and imaged using a confocal microscope.

Intracellular ROS levels were measured using a Reactive Oxygen Species Assay Kit (S0033S, Beyotime, China). Briefly, oocytes were incubated with 10 μM DCFH-DA probe in M2 media at 37 °C for 20 minutes. Subsequently, the oocytes were washed three times in DPBS supplemented with 0.1% BSA, mounted on glass-bottom Petri dishes, and observed under a laser scanning confocal microscope.

JC-1 MitoMP (MT09, Dojindo, Japan) was stained for potential-dependent accumulation in the mitochondria, characterized by a shift in fluorescence emission from green (~529 nm) to red (~590 nm). Briefly, oocytes were incubated with 2 μM JC-1 in M2 medium at 37 °C for 30 minutes, followed by washing with buffer for 10 minutes. Samples were immediately placed in a glass-bottom dish for imaging under a laser scanning confocal microscope.

To determine the intracellular glutathione level, oocytes were incubated in M2 medium containing 20 µM ThiolTracker™ Violet (T10096, Invitrogen) for 30 minutes at 37 °C. After three washes in M2 medium, oocytes were placed in glass-bottom Petri dishes and imaged with the LSM 880 confocal microscope system.

For detection of Fe^2+^ levels, oocytes were incubated with 1 μmol/L FerroOrange (F374, Dojindo, Japan) for 30 minutes in a 37 °C incubator equilibrated with 95% air and 5% CO_2_, and images were obtained on an LSM 880 laser scanning confocal microscope.

To evaluate lipid peroxidation, oocytes were incubated with M2 medium containing 10 μM BODIPYTM 581/591 C11 (D3861, Invitrogen) for 30 minutes in a 37 °C incubator equilibrated with 95% air and 5% CO_2_, and images were obtained on an LSM 880 laser scanning confocal microscope.

For quantification of fluorescence intensity, images from both control and treatment oocytes were acquired by a confocal microscope using the same parameters. ImageJ (NIH, Bethesda, MD, USA) was then applied to define a region of interest (ROI) in the image, and the mean fluorescence intensity per unit area within the ROI was determined. The mean values of all measurements were employed to facilitate a comparison of the final mean intensities between the control and treatment groups.

### 2.13 Statistics

Statistical analyses were performed using GraphPad Prism (version 9.0.0; GraphPad Software, San Diego, CA, USA). Five superovulated female mice were used to obtain oocytes for each experimental group in each independent batch. All experiments were repeated independently at least three times. Depending on the assay, 30–60 oocytes or embryos were analyzed per group in each batch. For developmental outcomes, including survival, fragmentation, fertilization (2PN), cleavage, morula, and blastocyst formation rates, percentages were calculated for each independent experimental batch, and these batch-level values were used for statistical analysis. For fluorescence-based analyses, fluorescence intensity was quantified in individual oocytes, with data obtained from at least three independent experiments. Normality and homogeneity of variances were assessed using the Shapiro–Wilk test and Brown–Forsythe test, respectively. Comparisons between two experimental groups were performed using an unpaired two-tailed Student’s t-test, whereas comparisons among three or more experimental groups were analyzed using one-way ANOVA followed by Tukey’s multiple comparisons test. Data are presented as the mean ± standard error of the mean (mean ± SEM). Statistical significance was defined as *P < 0.05, **P < 0.01, ***P < 0.001, and ****P < 0.0001 (ns, not significant).

## 3 Results

### 3.1 Vitrification reduced the developmental potential of oocytes

**Figure 1:**
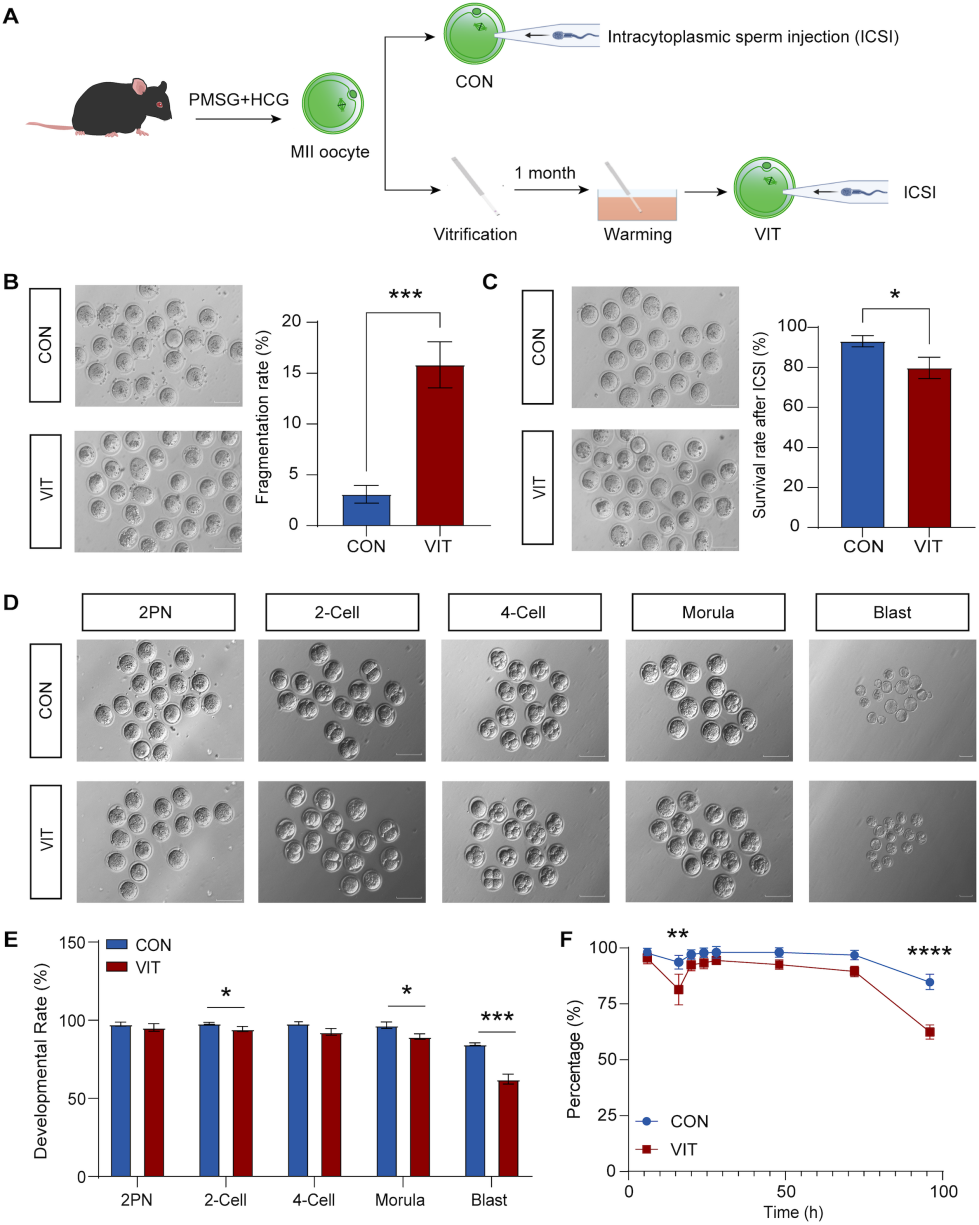
Vitrification reduced the developmental potential of oocytes. **(A)** Experimental diagram. **(B)** Fragmentation rates of oocytes in the CON and VIT groups. **(C)** Survival rates of oocytes following intracytoplasmic sperm injection (ICSI) in the CON and VIT groups. **(D)** Representative images of embryos cultured *in vitro* at 6, 24, 48, 72, and 96 h post-fertilization. Scale bar = 100 μm. **(E, F)** Developmental rates of early embryos at the 2-PN, 2-cell, 4-cell, morula, and blastocyst stages in the CON and VIT groups. Data are presented as mean ± SEM from at least three independent experiments, with >30 oocytes per group. Statistical comparisons were performed using Student’s t-test. *P < 0.05, **P < 0.01, ***P < 0.001, ****P < 0.0001.

We first established oocyte models of the fresh group (CON) and the vitrified group (VIT). The impact of vitrification on the development of early mouse embryos was investigated (**Figure 1A**). Following warming and 3 h of recovery culture in KSOM medium, the fragmentation rate of vitrified oocytes was significantly increased compared with the control group (*P* < 0.001) (**Figure 1B**). Six batches of vitrified oocytes demonstrated a recovery rate of over 90% (**Supplementary Figure 1A**). In order to mitigate the impact of recovery efficiency on embryonic development, subsequent experiments employed oocytes with a recovery rate of above 90%. Intracytoplasmic sperm injection (ICSI) was performed on recovered oocytes, and the post-ICSI survival rate was significantly lower in the VIT group than in the CON group (*P* < 0.05) (**Figure 1C**).

Although there was no significant difference in the fertilization rate of oocytes between the two groups (**Supplementary Figure 1B**), continuous *in vitro* culture indicated that oocytes in the CON group displayed higher 2-cell embryo, morula, and blastocyst formation rates (*P* < 0.05) compared to the VIT group (**Figures 1D, E**). In addition, the overall embryonic developmental rate was significantly reduced in the VIT group (*P* < 0.01) (**Figure 1F**). Embryo transfer via the oviduct using 2-cell stage embryos further demonstrated that the pregnancy rate in the VIT group was significantly lower than that in the CON group (*P* < 0.05) (**Supplementary Figure 1C**). These results demonstrate impaired developmental competence and reduced *in vivo* developmental potential following vitrification–warming.

### 3.2 Vitrification alters the glutathione metabolism in oocytes

**Figure 2:**
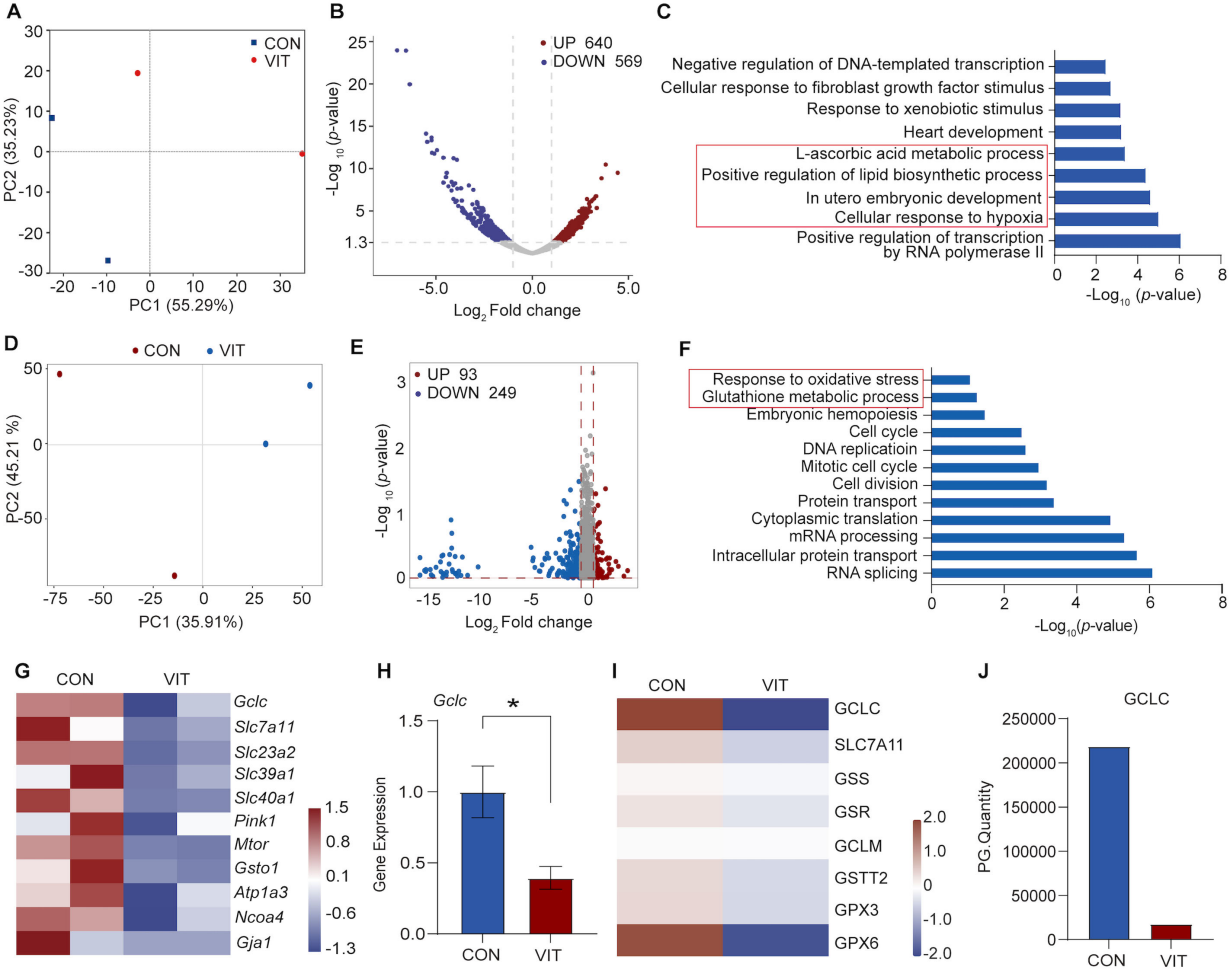
Vitrification alters the glutathione metabolism in oocytes. **(A)** Principal component analysis (PCA) of transcriptomic profiles of MII oocytes in the CON and VIT groups. **(B)** The volcano plot of differentially expressed genes (DEGs). **(C)** Gene Ontology (GO) enrichment analysis of down-regulated DEGs. **(D)** PCA of proteomic profiles of MII oocytes in the CON and VIT groups. **(E)** Volcano plot of differentially expressed proteins (DEPs). **(F)** GO enrichment analysis of down-regulated DEPs. **(G)** Heatmap of key genes enriched in the L-ascorbic acid metabolic process term. **(H)** Gene expression of Gclc in the CON and VIT groups. **(I)** Heatmap of the DEPs enriched in the glutathione metabolic process. **(J)** Protein expression levels of GCLC. Data are presented as mean ± SEM. Comparisons between experimental groups were analyzed by Student’s t-test, *P < 0.05.

RNA-seq analysis was performed on oocytes to identify the impact of vitrification on early embryonic development. A total of 1,209 differentially expressed genes (DEGs) were identified, applying the criteria of |fold change| > 2 and adjusted *P* value < 0.05, consisting of 640 upregulated genes and 569 downregulated genes (**Figures 2A, B**). These findings highlight the significant impact of vitrification on gene expression during early embryogenesis. Gene Ontology (GO) analysis revealed that the upregulated DEGs were enriched in pathways such as protein transport, negative regulation of transcription by RNA polymerase II, and negative regulation of mitochondrial cytochrome c release (**Supplementary Figure 2A**). Conversely, the downregulated DEGs were mainly enriched in pathways associated with positive regulation of transcription by RNA polymerase II, cellular response to hypoxia, intrauterine embryonic development, and L-ascorbic acid metabolism (**Figure 2C**).

Notably, several genes involved in L-ascorbic acid metabolism, including *Gclc*, *Slc7a11*, *Slc23a2*, *Gsto1*, and *Atp1a3*, showed altered expression in the VIT group (**Figure 2G**). L-ascorbic acid (vitamin C) is an important water-soluble antioxidant, and its metabolic process is associated with ferroptosis in multiple aspects (31, 32), including its role in antioxidant effects and the regulation of glutathione and iron metabolism (33–35). Glutathione (GSH) is a crucial intracellular antioxidant that plays a vital role in maintaining intracellular redox balance and resisting oxidative stress (36–38). Among these genes, *Gclc* emerged as a central regulator due to its role in catalyzing the rate-limiting step of glutathione synthesis (39–42). In the VIT group, the mRNA expression of *Gclc* was significantly decreased (*P* < 0.05) (**Figure 2H**).

We also compared the protein profiles between fresh and vitrified-warmed MII oocytes using micro-proteomics. Principal component analysis (PCA) revealed that the protein profiles of oocytes underwent significant changes after vitrification in comparison with the fresh oocytes (**Figure 2D**). As revealed by volcano plot analysis, 249 of these proteins were downregulated and 93 were upregulated (**Figure 2E**). In addition, GO analysis of the differentially expressed proteins (DEPs) indicated that compared with the fresh control group, the upregulated DEPs in vitrified-warmed oocytes were enriched in pathways such as negative regulation of release of cytochrome c from mitochondria and negative regulation of transcription by RNA polymerase II (**Supplementary Figure 2B**). The downregulated DEPs in vitrified-warmed oocytes were also highly enriched in glutathione metabolic processes and oxidative stress response pathways (**Figure 2F**). In particular, we observed a dramatic decrease in the protein level of GCLC—a key rate-limiting enzyme for glutathione synthesis—in oocytes after vitrification and warming (**Figures 2I, J**). Taken together, these findings indicate that the vitrification-warming process significantly disrupts the transcriptomic and proteomic profile of MII oocytes, particularly affecting pathways related to glutathione metabolism and oxidative stress. The pronounced reduction in GCLC further suggests that impaired glutathione synthesis may be a key mechanism underlying vitrification-induced oocyte damage.

### 3.3 Vitrification induced mitochondrial dysfunction and oxidative stress in oocytes

**Figure 3:**
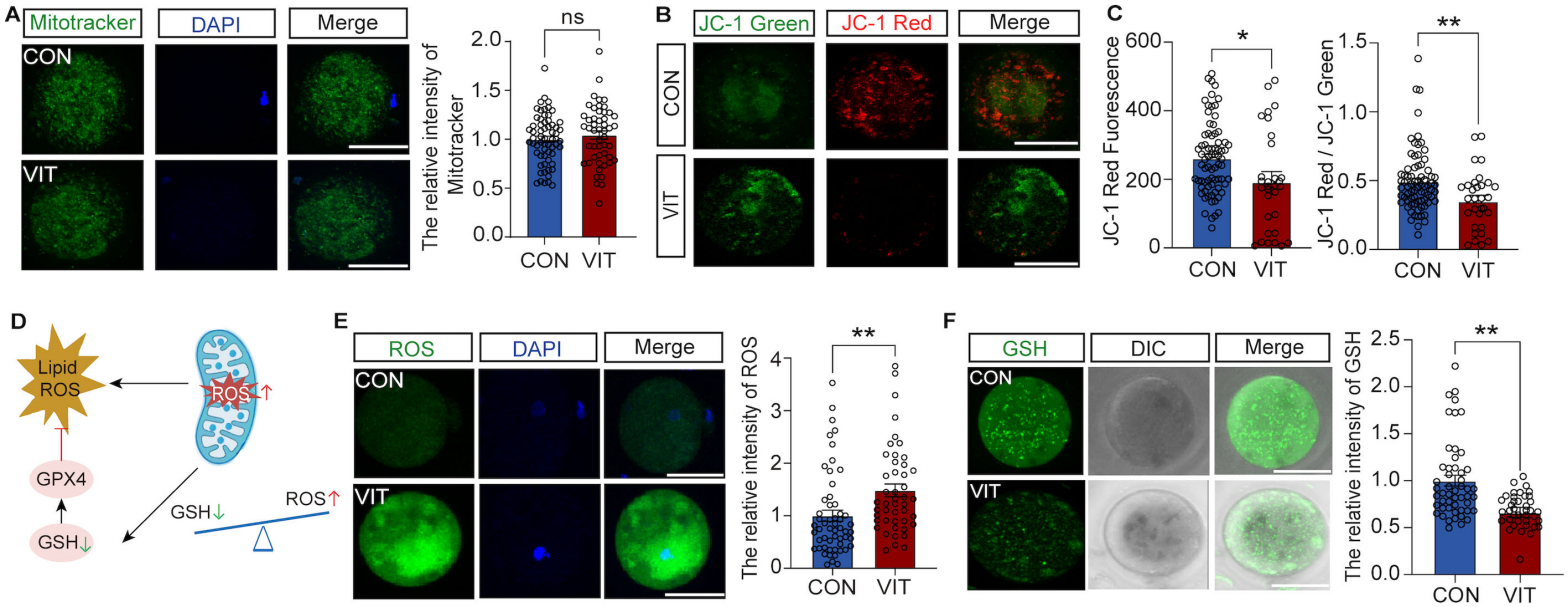
Vitrification induced mitochondrial dysfunction and oxidative stress in oocytes. **(A)** Representative images of mitochondrial distribution in MII oocytes from the CON and VIT groups. Scale bar = 50 μm. **(B)** Representative images of mitochondrial membrane potential levels (ΔΨm). Scale bar = 50 μm. **(C)** Representative images of JC-1 fluorescence intensity. Scale bar = 50 μm. **(D)** Schematic illustration of oxidative stress imbalance (ROS/GSH) in oocytes after vitrification. **(E)** Representative images of intracellular ROS levels. Scale bar = 50 μm. **(F)** Representative images of intracellular GSH levels. Scale bar = 50 μm. Data are presented as mean ± SEM from at least three independent experiments (>30 oocytes per group). Statistical comparisons were performed using Student’s t-test. *P < 0.05, **P < 0.01, and “ns” represents no significant difference.

Mitochondria are critical determinants of oocyte quality and play essential roles in oocyte maturation, fertilization, and embryonic development (43–45). To evaluate the effects of vitrification–warming on mitochondrial function, MitoTracker and JC-1 staining were performed. Although there was no significant difference in mitochondrial content in fresh and vitrified oocytes, the cellular mitochondrial distribution was markedly altered. In fresh oocytes, mitochondria were evenly distributed throughout the cytoplasm, whereas vitrified oocytes exhibited abnormal mitochondrial aggregation (**Figure 3A**). Mitochondrial membrane potential (ΔΨm) was evaluated using JC-1 staining. Red fluorescence indicates high ΔΨm, while green fluorescence indicates low ΔΨm. The red/green fluorescence intensity ratio was significantly decreased in vitrified oocytes compared with controls (*P* < 0.01) (**Figures 3B, C**). The findings indicate that vitrification–warming disrupts mitochondrial function in oocytes, leading to an imbalance between ROS production and intracellular GSH levels (**Figure 3D**).

Mitochondrial dysfunction has been shown to be associated with increased oxidative stress (45, 46), and excessive ROS can further exacerbate mitochondrial impairment (47, 48). The collection of oocytes was undertaken to facilitate an evaluation of the level of ROS. The results demonstrated that, in comparison with the control group, the ROS level in oocytes following vitrification was considerably elevated (*P* < 0.01) (**Figure 3E**). In contrast, intracellular glutathione (GSH) levels were significantly reduced following vitrification and warming (*P* < 0.01) (**Figure 3F**). Collectively, these findings provide further evidence that vitrification increases oxidative stress and reduces intracellular glutathione content in oocytes, indicating a disruption of the oxidative–antioxidative balance.

### 3.4 Vitrification increased lipid peroxidation and ferroptosis in oocytes

**Figure 4:**
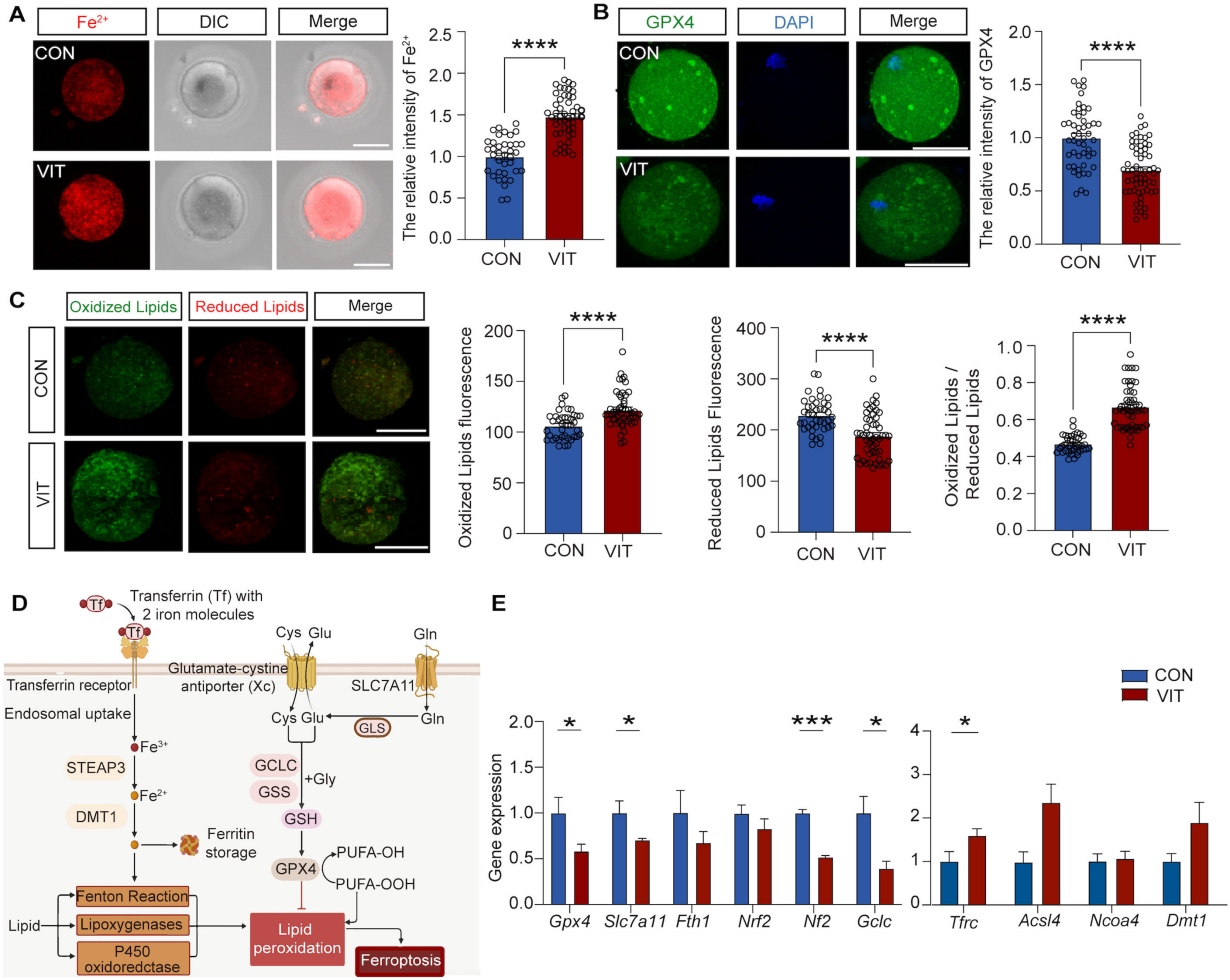
Vitrification induces ferroptosis and lipid peroxidation in oocytes. **(A)** Representative images of intracellular Fe^2+^ levels in MII oocytes from the CON and VIT groups. Scale bar = 50 μm. **(B)** Representative images of GPX4 immunofluorescence signals. Scale bar = 50 μm. **(C)** Representative images of lipid peroxidation levels. Scale bar = 50 μm. **(D)** Schematic diagram illustrating the disruption of ferroptosis-related pathways following vitrification. **(E)** Relative mRNA expression levels of ferroptosis-related genes. Data are presented as mean ± SEM from at least three independent experiments (>30 oocytes per group). Statistical comparisons were performed using Student’s t-test. *P < 0.05, ***P < 0.001, ****P < 0.0001.

Ferroptosis is characterized by excessive reactive oxygen species (ROS) production, depletion of glutathione (GSH), and increased lipid peroxidation (20). It has been reported that adequate GSH can serve as a substrate for glutathione peroxidase (GPX), thereby enabling GPX to promptly scavenge intracellular lipid peroxides and inhibit ferroptosis (**Figure 4D**). To assess ferroptosis-related changes, intracellular ferrous iron (Fe^2+^) levels were measured using the fluorescent probe FerroOrange. The results showed that Fe^2+^ levels were significantly elevated in vitrified–warmed oocytes compared with fresh controls (*P* < 0.0001) (**Figure 4A**).

Given that disrupted iron homeostasis can induce lipid peroxidation, we further evaluated lipid peroxidation levels in oocytes. Through BODIPY-C11 581/591 staining, we found that the level of lipid peroxidation in oocytes was significantly elevated (*P* < 0.0001) after vitrification (**Figure 4C**). In addition, the protein expression of GPX4 was examined by immunostaining. GPX4 levels were markedly reduced in vitrified–warmed oocytes compared with the control group (*P* < 0.0001) (**Figure 4B**). Furthermore, analysis of ferroptosis-related gene expression revealed that *Slc7a11*, *Gclc*, and *Gpx4* were significantly downregulated in the vitrification (VIT) group (*P* < 0.05) (**Figure 4E**). Together, these results demonstrate that vitrification–warming induces ferroptosis-related alterations in oocytes.

### 3.5 RSL3 induces ferroptosis-associated changes in mouse oocytes

**Figure 5:**
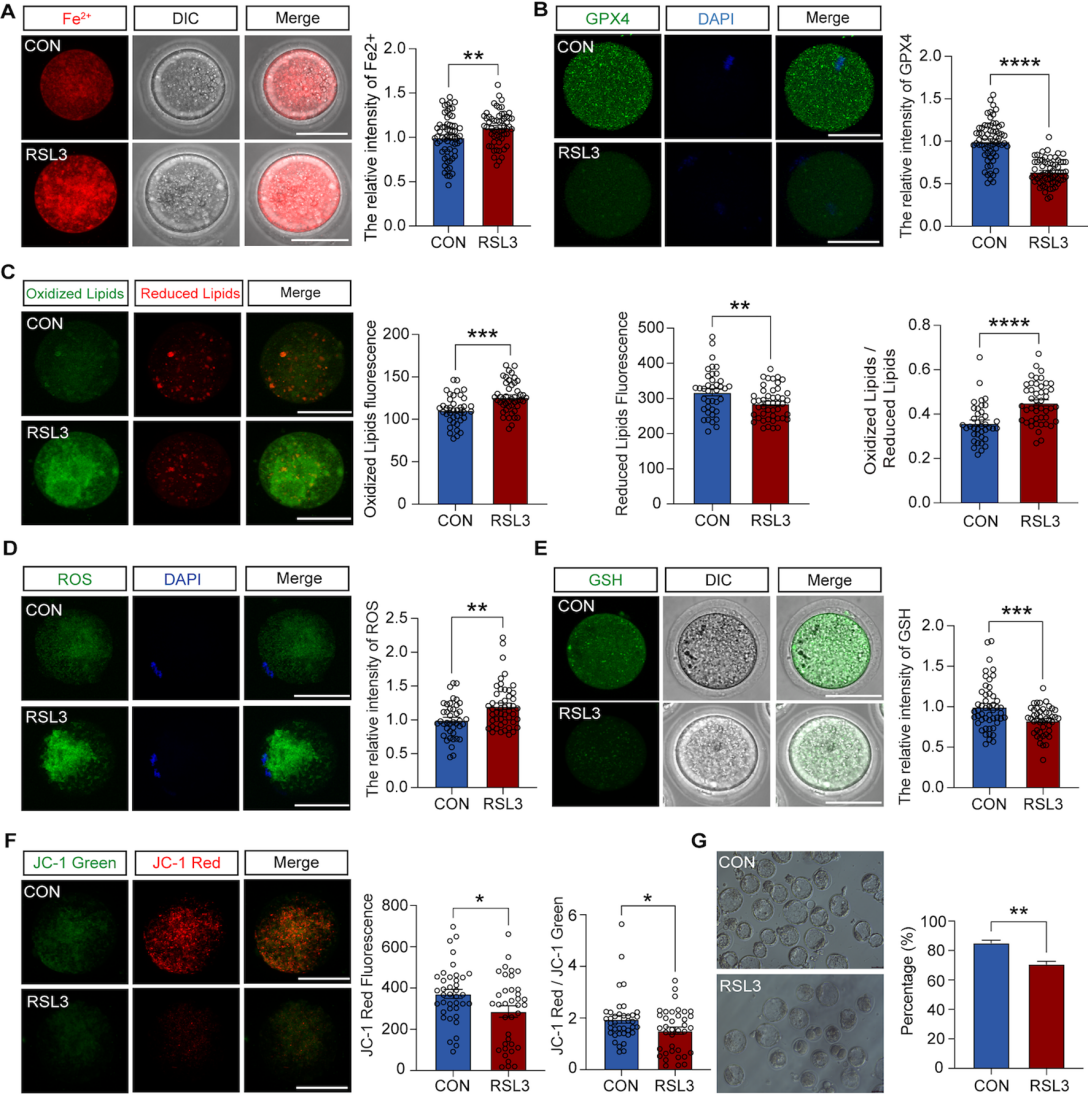
RSL3 induces ferroptosis-associated changes in mouse oocytes. **(A)** Representative images of intracellular Fe^2+^ levels in MII oocytes from the CON and RSL3-treated groups. Scale bar = 50 μm. **(B)** Representative images of GPX4 immunofluorescence signals. Scale bar = 50 μm. **(C)** Representative images of lipid peroxidation levels. Scale bar = 50 μm. **(D)** Representative images of intracellular ROS levels. Scale bar = 50 μm. **(E)** Representative images of intracellular GSH levels. Scale bar = 50 μm. **(F)** Representative images of JC-1 fluorescence intensity. Scale bar = 50 μm. **(G)** Representative images of blastocysts and quantification of blastocyst formation rates. Scale bar = 50 μm. Data are presented as mean ± SEM from at least three independent experiments (>30 oocytes per group). Statistical comparisons were performed using Student’s t-test. *P < 0.05, **P < 0.01, ***P < 0.001, ****P < 0.0001.

To further assess the involvement of ferroptosis, fresh MII-stage oocytes were treated with RSL3, a selective inhibitor of GPX4 and a widely used inducer of ferroptosis. RSL3 treatment increased intracellular ROS levels, Fe^2+^ accumulation, and lipid peroxidation, and was accompanied by a reduction in GPX4 immunofluorescence intensity and intracellular GSH levels, and mitochondrial membrane potential (**Figures 5A–F**). These changes were associated with impaired oocyte quality and reduced subsequent embryonic development, with a marked decrease in blastocyst formation compared with the control group (**Figure 5G**). Together, these results suggest that RSL3 induces changes similar to those observed after vitrification-warming.

### 3.6 Fer-1 and GSH-MEE inhibit ferroptosis in oocytes induced by vitrification

**Figure 6:**
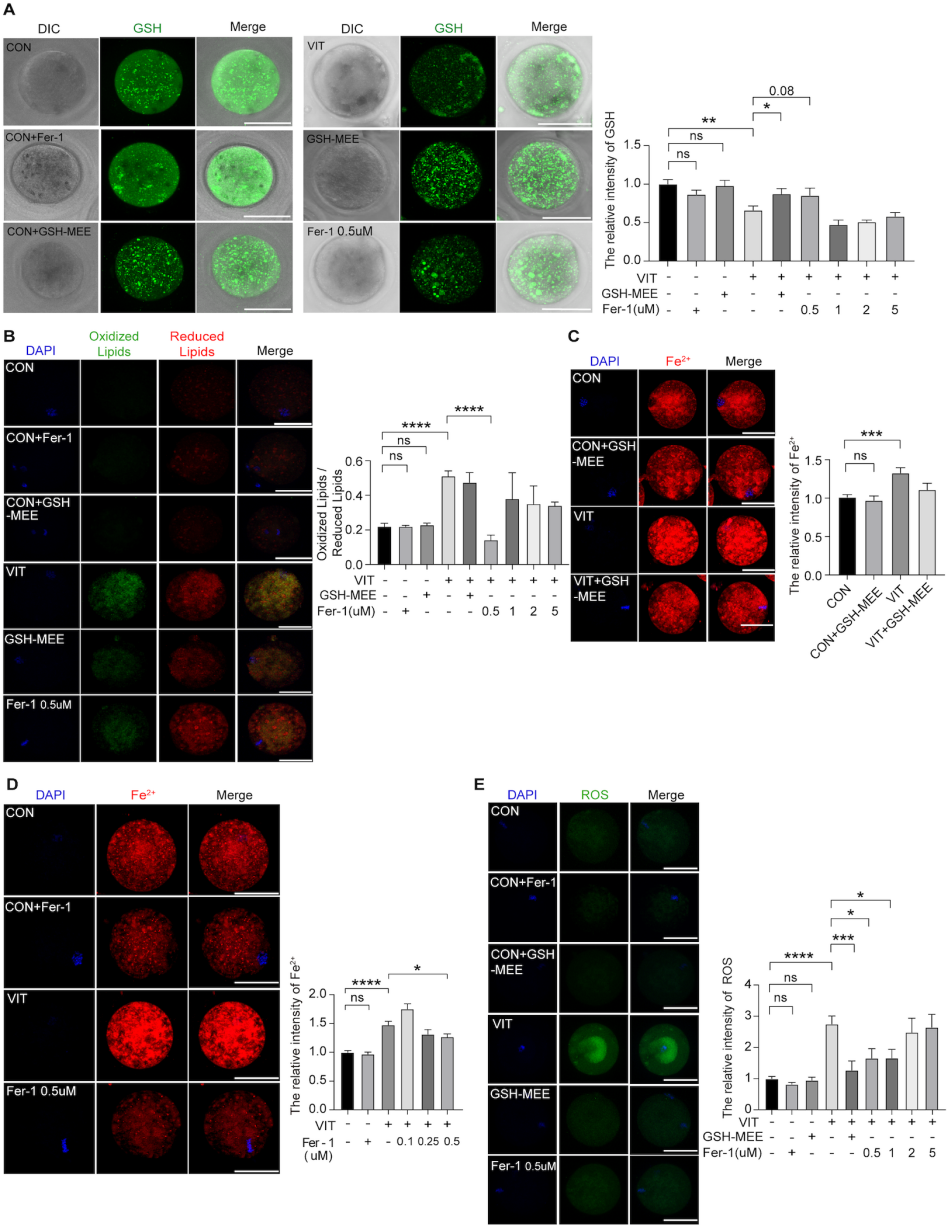
Fer-1 and GSH-MEE inhibit ferroptosis in vitrified oocytes. **(A)** Representative images of GSH levels from MII oocytes in CON, CON+Fer-1, CON+GSH-MEE (0.5 mM), VIT, VIT+GSH-MEE (0.5 mM), and VIT+Fer-1 groups. Scale bar = 50 μm. **(B)** Representative images of lipid peroxidation levels. Scale bar = 50 μm. **(C, D)** Representative images of intracellular Fe^2+^ levels. Scale bar = 50 μm. **(E)** Representative images of intracellular ROS levels. Scale bar = 50 μm. Data are presented as mean ± SEM from at least three independent experiments (>30 oocytes per group). Statistical comparisons were performed using one-way ANOVA. *P < 0.05, ***P < 0.001, ****P < 0.0001.

Previous studies have shown that RSL3-induced ferroptosis can be reversed by ferroptosis inhibitors, such as deferoxamine mesylate (DFO), ferrostatin-1 (Fer-1), and vitamin E (VE) (49–52). In this study, Fer-1 and GSH-MEE were applied to vitrified–warmed oocytes to evaluate their effects. Following vitrification–warming, oocytes were cultured for 3 h in medium supplemented with Fer-1 or GSH-MEE. The concentrations used (0.5 mM GSH-MEE and a range of Fer-1 concentrations) were selected based on previous reports (24). Measurement of intracellular GSH levels showed that both Fer-1 and GSH-MEE treatments increased GSH content in vitrified–warmed oocytes (**Figure 6A**). Furthermore, quantitative analysis of Fe^2+^, reactive oxygen species (ROS), and lipid peroxidation demonstrated that supplementation with 0.5 mM GSH-MEE or 0.5 μM Fer-1 significantly reduced iron accumulation, oxidative stress, and lipid peroxidation in vitrified oocytes (*P* < 0.05) (**Figures 6B–E**). To examine whether Fer-1 and GSH-MEE affected non-vitrified oocytes, we also included CON + Fer-1 and CON + GSH-MEE groups. As shown in the analysis, these treatments did not lead to obvious changes in GSH levels, Fe^2+^ content, ROS production, or lipid peroxidation compared with untreated controls (**Figures 6B–E**), suggesting that Fer-1 and GSH-MEE had no detectable effect on non-vitrified oocytes.

### 3.7 Inhibition of ferroptosis restores the developmental competence of vitrified oocytes

**Figure 7:**
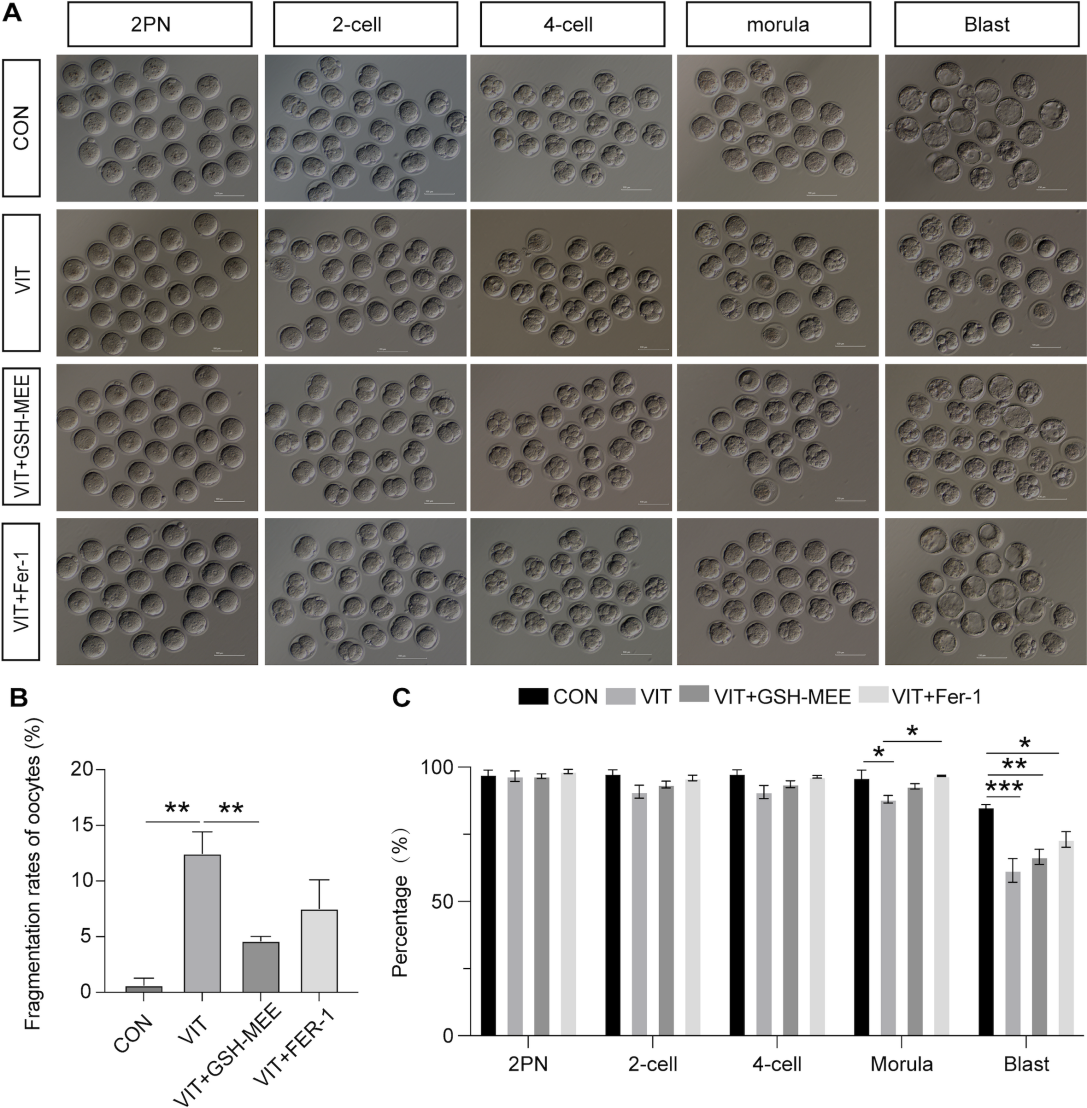
Inhibition of ferroptosis restores the developmental competence of vitrified oocytes. **(A)** Representative images of mouse embryos derived from MII oocytes in CON, VIT, VIT+GSH-MEE, and VIT+Fer-1 groups *in vitro* cultured for 6 h, 24 h, 48 h, 72 h, and 96 h post-fertilization. Scale bar = 100 μm. **(B)** Fragmentation rates of oocytes in each group. **(C)** Developmental rates of early embryos at the 2-PN, 2-cell, 4-cell, morula, and blastocyst stages. Data are presented as mean ± SEM from at least three independent experiments (>30 oocytes per group). Statistical comparisons were performed using one-way ANOVA. *P < 0.05, **P < 0.01, ***P < 0.001.

Oocyte quality is critical for fertilization and subsequent embryonic development. To assess the effects of ferroptosis inhibition, intracytoplasmic sperm injection (ICSI) was performed using vitrified–warmed oocytes. Oocyte fragmentation was assessed 3 h after warming. Supplementation with GSH-MEE significantly reduced the fragmentation rate compared with the vitrification (VIT) group. A decreasing trend was also observed in the Fer-1-treated group (*P* < 0.01) (**Figure 7B**).

Although there was no significant difference in the 2PN formation rate, the 2-cell embryo formation rate of the VIT group showed a slight improvement after supplementation with GSH-MEE or Fer-1 in the culture medium. In addition, both GSH-MEE and Fer-1 significantly increased the blastocyst formation rate (*P* < 0.05) (**Figures 7A, C**). These results suggest that inhibition of ferroptosis is a potential approach to reverse the decline in oocyte embryonic developmental potential that occurs following vitrification and warming.

## 4 Discussion

In this study, we identify ferroptosis as a previously underappreciated mechanism contributing to oocyte damage following vitrification–warming. To better mimic clinical conditions, mouse MII-stage oocytes were obtained via ovulation induction and used to establish a vitrified–warmed oocyte model. We found that vitrification-warming increased the rate of oocyte fragmentation and reduced oocyte survival following intracytoplasmic sperm injection (ICSI), consistent with previous reports (13, 53, 54). Furthermore, embryos derived from vitrified–warmed oocytes exhibited pronounced developmental delay, characterized by increased 2-cell-stage arrest and reduced blastocyst formation rates. Notably, this phenotype is not restricted to our model but has been consistently observed across multiple species and experimental systems. Studies in human embryos have shown that although most embryos survive warming, their developmental competence—reflected by high-quality embryo rates, cleavage kinetics, and blastocyst formation—remains compromised and is often accompanied by reduced morphological scores and chromosomal abnormalities (55–58). Similar observations have been made in mice, where vitrified oocytes show acceptable fertilization rates but produce embryos with reduced developmental potential and lower live birth rates (13, 24, 54). In porcine models, vitrification has been shown to delay embryonic genome activation, a key checkpoint for subsequent development (59–62). Collectively, these findings suggest that impaired developmental competence is a conserved outcome of oocyte vitrification, underscoring the need for targeted interventions.

To explore the underlying mechanisms, we performed integrated transcriptomic and proteomic analyses. These data revealed a marked reduction in glutathione (GSH) levels, a key substrate of GPX4. The Xc^−^–GSH–GPX4 axis constitutes a central antioxidant system regulating ferroptosis, and involves transporters such as SLC7A11 and SLC3A2 (63, 64). This system maintains intracellular GSH in a reduced state (20, 63). As the principal intracellular antioxidant, GSH reduces lipid peroxides to less toxic alcohols, thereby limiting lipid peroxidation damage (65, 66). Suppression of GSH is a hallmark of ferroptosis and is closely associated with GPX4 activity (67–69). In addition, glutamate–cysteine ligase catalytic subunit (GCLC), which catalyzes the rate-limiting step in GSH synthesis, has been shown to inhibit ferroptosis. In our study, GCLC was significantly downregulated at both the mRNA and protein levels, accompanied by altered SLC7A11 expression and a marked depletion of intracellular GSH in vitrified–warmed oocytes. Together, these findings indicate that vitrification–warming disrupts the Xc^−^–GSH–GPX4 antioxidant system and is associated with ferroptosis-related alterations, suggesting that disruption of the Xc^−^–GSH–GPX4 antioxidant system may contribute to the decline in oocyte and embryonic quality following cryopreservation.

Ferroptosis, first described in 2012, is a form of regulated cell death characterized by excessive reactive oxygen species (ROS) production and iron-dependent lipid peroxidation (70). Previous studies have shown that ferric ammonium citrate (FAC), a ferroptosis inducer, can trigger mitochondrial dysfunction and ferroptosis in porcine oocytes, leading to meiotic arrest and reduced embryonic cleavage and blastocyst formation rates (71). Consistent with these findings, we observed mitochondrial dysfunction, including abnormal mitochondrial distribution and decreased membrane potential, accompanied by elevated ROS levels in vitrified oocytes. Increased Fe^2+^ accumulation, enhanced lipid peroxidation, reduced GPX4 expression, and GSH depletion are consistent with ferroptosis-associated changes. These findings suggest that ferroptosis-related processes may contribute to the relationship between oxidative stress and mitochondrial dysfunction. Importantly, our results suggest that pharmacological inhibition of ferroptosis can partially alleviate these alterations. Supplementation with Fer-1 or GSH-MEE reduced oxidative stress, iron overload, and lipid peroxidation, while restoring intracellular GSH levels. Notably, these treatments did not significantly alter 2PN formation rates, but partially improved subsequent embryonic development, particularly blastocyst formation. These observations are consistent with previous studies demonstrating that ferroptosis inhibitors, such as vitamin E, deferoxamine, and liproxstatin-1, can rescue cellular dysfunction in various models (50–52).

Emerging evidence further supports the role of ferroptosis in oocyte quality regulation. For instance, recessive mutations in COX15, a component of mitochondrial respiratory chain complex IV, contribute to oocyte degeneration via ferroptosis pathways (44). Inhibition of ferroptosis has also been shown to rescue oocyte degeneration associated with advanced maternal age (72). Moreover, MIT-001 improves embryo quality by alleviating ferroptosis-associated mitochondrial dysfunction in aged mice (28), while melatonin suppresses ferroptosis through Nrf2 signaling in porcine oocytes (73). Together with our findings, these studies support a potential role for ferroptosis-related pathways in regulating oocyte competence and early embryonic development.

In conclusion, our study shows that vitrification-warming is associated with ferroptosis-related alterations in mouse oocytes, including increased oxidative stress, lipid peroxidation, iron accumulation, and disruption of the GSH–GPX4 antioxidant system. Treatment with Fer-1 or GSH-MEE partially improved oocyte quality and subsequent embryonic development after cryopreservation. These findings suggest that ferroptosis-related pathways may be involved in vitrification-warming-induced oocyte injury.

However, this study was performed exclusively in mouse oocytes, and further studies using human oocytes will be needed to evaluate the clinical relevance of these findings. Although mouse models are widely used to investigate mechanisms associated with oocyte vitrification-warming injury, species-specific differences in oocyte physiology, metabolism, and sensitivity to cryopreservation stress may influence the applicability of these results to humans. In addition, the transcriptomic and proteomic analyses were based on two biological replicates per group. The results were consistent with our biochemical and functional data and helped identify pathways associated with vitrification-warming. Given the limited number of replicates, these findings should be considered supportive evidence and require further validation. Finally, the long-term safety of ferroptosis-targeted interventions, including their potential effects on embryo and offspring development, requires further evaluation before clinical application.

## Data Availability

The datasets presented in this study can be found in online repositories. The names of the repository/repositories and accession number(s) can be found in the article/**Supplementary Material**.
