# Supplementary material for: Inhibition of ferroptosis improves developmental competence of vitrified–warmed oocytes
Source: Front Endocrinol (Lausanne). 2026 Jun 15;17:1851814. doi: 10.3389/fendo.2026.1851814 (PMC13310709; doi:10.3389/fendo.2026.1851814)
Supplement: Supplementary file 1 [file DataSheet1.docx]

Supplementary Material

# Supplementary Data

Figure S1: Vitrification reduced the developmental potential of oocytes; Figure S2: Vitrification alters the glutathione metabolism in oocytes; Table S1: Primers used for real-time quantitative PCR; Table S2: Proteomics of oocytes.

# Supplementary Figures and Tables

## Supplementary Figures


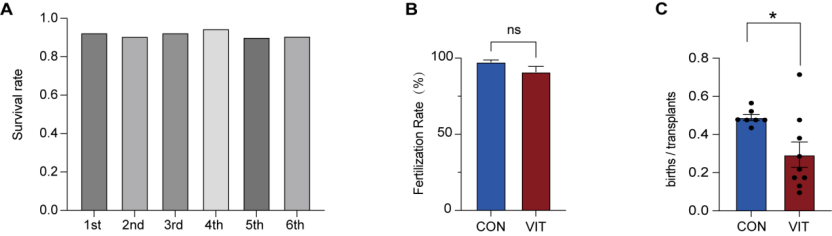


**Supplementary Figure 1.** **Vitrification reduces the developmental potential of oocytes.**

(A) Recovery rates of oocytes in the VIT group. (B) Fertilization rates of oocytes in the CON and VIT group (three independent experiments, with >30 oocytes per group). (C) Pregnancy rates in the CON and VIT groups. Data are presented as mean ± SEM from at least three independent experiments. Statistical comparisons between groups were performed using a two-tailed Student’s t-test. **P* < 0.05, and "ns" represents no significant difference.


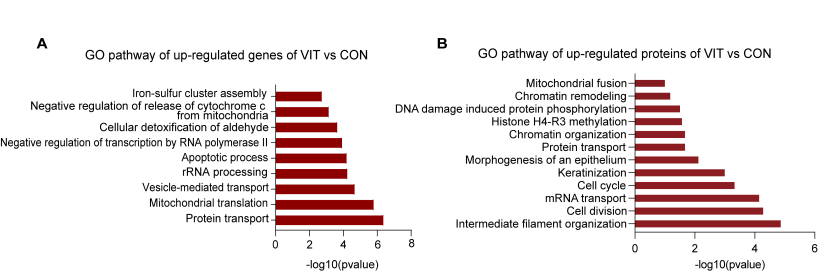


**Supplementary Figure 2. Vitrification alters glutathione metabolism in oocytes.**

1. Gene Ontology (GO) enrichment analysis of upregulated differentially expressed genes (DEGs) in the CON and VIT groups. (B) GO enrichment analysis of upregulated differentially expressed proteins (DEPs) in the CON and VIT groups.

**
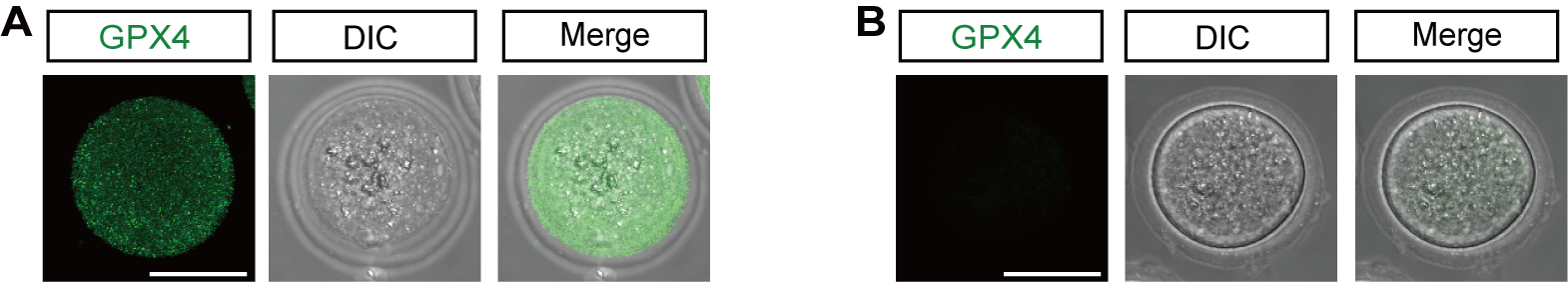
**

**Supplementary Figure 4. Vitrification induces ferroptosis and lipid peroxidation in oocytes.**

(A) Representative images of GPX4 immunofluorescence staining in mouse oocytes. (B) Representative images of isotype-matched IgG controls. No specific fluorescence signal was observed in the IgG control group. Scale bar = 50 μm.
