# Supplementary material for: Inhibition of ferroptosis improves developmental competence of vitrified–warmed oocytes
Source: Front Endocrinol (Lausanne). 2026 Jun 15;17:1851814. doi: 10.3389/fendo.2026.1851814 (PMC13310709; doi:10.3389/fendo.2026.1851814)
Supplement: Supplementary file 2 [file DataSheet2.doc]

Supplementary Table 1. Primers used for real-time quantitative PCR

| Gene | Accession No. | Primer direction | Sequences (5’to 3’) | Product Size (bp) |
| --- | --- | --- | --- | --- |
| *Gclc* | [NM_010295.2](https://www.ncbi.nlm.nih.gov/entrez/viewer.fcgi?db=nucleotide&id=324710985) | Forward | GAAGGGGTATTTCCTGGACTCATC | 154 |
|  |  | Reverse | CTCTCATCCACCTGGCAACAGT |  |
| *Slc7a11* | [NM_011990.2](https://www.ncbi.nlm.nih.gov/entrez/viewer.fcgi?db=nucleotide&id=80861466) | Forward | CTATTTTACCACCATCAGTGCG | 102 |
|  |  | Reverse | ATCGGGACTGCTAATGAGAATT |  |
| *Fth1* | [NM_010239.2](https://www.ncbi.nlm.nih.gov/entrez/viewer.fcgi?db=nucleotide&id=407027852) | Forward | TAAAGAAACCAGACCGTGATGA | 80 |
|  |  | Reverse | ATTCACACTCTTTTCCAAGTGC |  |
| *Gpx4* | [NM_001037741.4](https://www.ncbi.nlm.nih.gov/entrez/viewer.fcgi?db=nucleotide&id=1547242142) | Forward | CCTCCCCAGTACTGCAACAG | 93 |
|  |  | Reverse | GGCTGAGAATTCGTGCATGG |  |
| *Nrf2* | [NM_010902.5](https://www.ncbi.nlm.nih.gov/entrez/viewer.fcgi?db=nucleotide&id=2199407475) | Forward | TCCTATGCGTGAATCCCAAT | 103 |
|  |  | Reverse | GCGGCTTGAATGTTTGTCTT |  |
| *Nf2* | [NM_001361676.1](https://www.ncbi.nlm.nih.gov/entrez/viewer.fcgi?db=nucleotide&id=1371986095) | Forward | GAGGAGAGAATTACTGCTTGGT | 134 |
|  |  | Reverse | CCCTTTTTATTCCGGATTGCAA |  |
| *Tfrc* | [NM_011638.4](https://www.ncbi.nlm.nih.gov/entrez/viewer.fcgi?db=nucleotide&id=291045184) | Forward | TCACACTCTCTCAGCTTTAGTG | 84 |
|  |  | Reverse | TGGTTTCTGAAGAGGGTTTCAT |  |
| *Acsl4* | [NM_001033600.1](https://www.ncbi.nlm.nih.gov/entrez/viewer.fcgi?db=nucleotide&id=75992924) | Forward | CAATAGAGCAGAGTACCCTGAG | 146 |
|  |  | Reverse | TAGAACCACTGGTGTACATGAC |  |
| *Ncoa4* | [NM_001403005.1](https://www.ncbi.nlm.nih.gov/entrez/viewer.fcgi?db=nucleotide&id=2211517518) | Forward | ACCAGCCTAGAGGTGTGGAGATTG | 134 |
|  |  | Reverse | GTCCTGATGGTTCTGGGCAAGC |  |
| *Dmt1* | [NM_001146161.1](https://www.ncbi.nlm.nih.gov/entrez/viewer.fcgi?db=nucleotide&id=225903447) | Forward | TGAATCGGGCCAATAAGCAGGA | 118 |
|  |  | Reverse | TCAGCAAAGACGGACACGACAA |  |
| *Gapdh* | [NM_001289746.2](https://www.ncbi.nlm.nih.gov/entrez/viewer.fcgi?db=nucleotide&id=1675059531) | Forward | CAGGAGGCATTGCTGATGAT | 138 |
|  |  | Reverse | GAAGGCTGGGGCTCATTT |  |
